# Insights into the neutral and adaptive processes shaping the spatial distribution of genomic variation in the economically important Moroccan locust (*Dociostaurus maroccanus*)

**DOI:** 10.1002/ece3.6165

**Published:** 2020-03-31

**Authors:** María José González‐Serna, Pedro J. Cordero, Joaquín Ortego

**Affiliations:** ^1^ Grupo de Investigación de la Biodiversidad Genética y Cultural Instituto de Investigación en Recursos Cinegéticos – IREC – (CSIC, UCLM, JCCM) Ciudad Real Spain; ^2^ Departamento de Ciencia y Tecnología Agroforestal y Genética Escuela Técnica Superior de Ingenieros Agrónomos (ETSIA) Universidad de Castilla‐La Mancha (UCLM) Ciudad Real Spain; ^3^ Department of Integrative Ecology Estación Biológica de Doñana – EBD – (CSIC) Seville Spain

**Keywords:** ddRadSeq, demographic inference, environmental association analyses, genetic structure, local adaptation, pest species

## Abstract

Understanding the processes that shape neutral and adaptive genomic variation is a fundamental step to determine the demographic and evolutionary dynamics of pest species. Here, we use genomic data obtained via restriction site‐associated DNA sequencing to investigate the genetic structure of Moroccan locust (*Dociostaurus maroccanus*) populations from the westernmost portion of the species distribution (Iberian Peninsula and Canary Islands), infer demographic trends, and determine the role of neutral versus selective processes in shaping spatial patterns of genomic variation in this pest species of great economic importance. Our analyses showed that Iberian populations are characterized by high gene flow, whereas the highly isolated Canarian populations have experienced strong genetic drift and loss of genetic diversity. Historical demographic reconstructions revealed that all populations have passed through a substantial genetic bottleneck around the last glacial maximum (~21 ka BP) followed by a sharp demographic expansion at the onset of the Holocene, indicating increased effective population sizes during warm periods as expected from the thermophilic nature of the species. Genome scans and environmental association analyses identified several loci putatively under selection, suggesting that local adaptation processes in certain populations might not be impeded by widespread gene flow. Finally, all analyses showed few differences between outbreak and nonoutbreak populations. Integrated pest management practices should consider high population connectivity and the potential importance of local adaptation processes on population persistence.

## Graphical abstract

In this study, we use genomic data (ddRADseq) to infer historical demographic trends and determine the relative role of neutral versus selective processes on shaping spatial patterns of genomic variation in the Moroccan locust, a pest species of great economic importance. Our study revealed low levels of genetic differentiation at regional scales, signatures of divergent selection in several loci, and a demographic history characterized by peaks of effective population sizes matching with warm periods (interglacials) during the Pleistocene. All our analyses also showed very little differences between traditionally outbreak and nonoutbreak populations in patterns of genomic diversity and structure, demographic profiles, or signatures of selection. Overall, our study suggests that integrated management practices aimed at reducing the negative impacts of this locust species on agriculture should consider population connectivity at contrasting spatial scales, the potential role of local adaptation processes on population persistence and likely future range expansions, and increased outbreak frequency in response to ongoing climate warming.

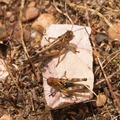

## 1 INTRODUCTION

The genetic makeup of a species is influenced by complex interactions between neutral and selective forces, life‐history characteristics, and contemporary and past environmental conditions that collectively shape the evolutionary and demographic fate of its populations (Bernatchez et al., 2018). From a neutral perspective, recently developed analytical methods allow inferring complex demographic processes from genomic data and obtaining robust estimates of population size changes and gene flow at different time scales (Liu & Fu, 2015; Miles et al., 2017; Sherpa et al., 2018). Historical demographic reconstructions, linking population size changes with past environmental fluctuations, can help to predict future demographic trends of species under hypothetical climate change scenarios (Brown et al., 2016; Espindola et al., 2012; Fordham, Brook, Moritz, & Nogués‐Bravo, 2014), whereas contemporary estimates of population genetic connectivity and spatially explicit landscape genetic analyses are useful to obtain baseline information on dispersal rates (Crossley, Rondon, & Schoville, 2019a) and identify corridors for gene flow (Crossley, Rondon, & Schoville, 2019b; Karsten, Addison, Jansen van Vuuren, & Terblanche, 2016; Qin et al., 2018; Venkatesan & Rasgon, 2010; Zepeda‐Paulo et al., 2010). From an adaptive perspective, new molecular tools bring the opportunity to infer selection at specific loci displaying patterns of variation contrasting to those characterizing genomic regions only affected by neutral processes such as mutation, genetic drift, migration, and demographic changes (Berdan, Mazzoni, Waurick, Roehr, & Mayer, 2015; Luikart, England, Tallmon, Jordan, & Taberlet, 2003). Identifying loci that exhibit a significant reduction of within‐population genetic variability and higher divergence across populations consistent with disruptive selection (Vasemägi & Primmer, 2005), coupled with environment association analyses (EAA) linking variation in allele frequencies with ecological gradients (Rellstab, Gugerli, Eckert, Hancock, & Holderegger, 2015), can help to determine local adaptation processes in response to specific selective forces such as those imposed by climate (Crossley, Chen, Groves, & Schoville, 2017; Dowle et al., 2017; Dudaniec, Yong, Lancaster, Svensson, & Hansson, 2018; Guo, Li, & Merilä, 2016), pesticide application (Crossley et al., 2017; Gassmann, Onstad, & Pittendrigh, 2009; Leftwich, Bolton, & Chapman, 2016), or host plant use (Gassmann et al., 2009; Simon et al., 2015; Soria‐Carrasco et al., 2014). Gene flow is generally accepted to constrain local adaptation whereas strong divergent selection is expected to prevent interpopulation realized gene flow (Lenormand, 2002). Thus, joined inference of both neutral and selective phenomena can provide a more comprehensive understanding on the relative role of dispersal and local adaptation processes on structuring genetic variation (Dudaniec et al., 2018; Guo et al., 2016).

Pest species, either invasive or native, are responsible of considerable economic losses worldwide and, as a result, public, private, and nonprofit organizations annually invest vast amounts of resources to prevent or mitigate their negative impacts (Enserink, 2004; Skaf, Popov, Roffey, Scorer, & Hewitt, 1990). In turn, such management practices often have undesirable side effects on wildlife, natural ecosystems, and human health (e.g., Baker & Wilkinson, 1990; Carson, 2002). For these reasons, understanding the population dynamics, dispersal routes, demographic history, and idiosyncratic evolutionary processes of pest species is a fundamental step to predict their future impacts and develop informed, less pernicious, and more targeted management practices (Abrol, 2014; Lankau, Jørgensen, Harris, & Sih, 2011). Population genetic approaches have proven useful to address several of the abovementioned aspects, and their potential has exponentially grown in the last years by the generalization of high‐throughput sequencing techniques and the possibility of inferring both neutral and adaptive evolutionary processes at an unprecedented resolution (Crossley et al., 2017; Wang et al., 2014). The application of genomic tools is particularly important if we consider that most pest species often show large effective population sizes, high dispersal potential, shallow genetic differentiation, and fluctuating and complex demographic dynamics that are difficult to study using traditional capture–mark–recapture approaches or standard genetic methods (Bekkevold, Gross, Arula, Helyar, & Ojaveer, 2016; Chapuis et al., 2008, 2011; Ibrahim, Sourrouille, & Hewitt, 2000; Kirk, Dorn, & Mazzi, 2013).

Locusts are a paradigmatic case of pest species with cyclical outbreaks that cause considerable agricultural losses and remission periods during which local populations either disappear or persist at very low densities (Chapuis et al., 2009; Enserink, 2004; Latchininsky, 1998; Skaf et al., 1990). The prevalence of outbreak and solitary phases varies geographically, with populations from some areas recurrently becoming agricultural pests while those from nonoutbreaking regions often occur at low numbers forming harmless populations (Chapuis et al., 2009; Latchininsky, 1998). An intriguing example of this demographic stochasticity is the case of the mysterious Rocky Mountain locust *Melanoplus spretus*, a devastating pest species endemic from North American prairies during the 19th century that went inexplicably extinct within a 30‐year interval (Chapco & Litzenberger, 2004; Lockwood, 2004). Extreme demographic oscillations charactering locust populations are expected to have a considerable impact on genetic variation and the potential of species to respond to selection and evolve local adaptations. On the one hand, population crashes in the transition phase from gregarious to solitary forms are likely to leave genetic signatures of demographic bottlenecks that are probably ephemeral and blurred by genetic admixture after population expansions during outbreak periods (Chapuis et al., 2008; Ibrahim, 2001; Ibrahim et al., 2000). On the other hand, local adaptation processes in response to spatially varying selective pressures could be impeded by high gene flow (Babin, Gagnaire, Pavey, & Bernatchez, 2017; Lenormand, 2002; Pujolar et al., 2014) or restricted to isolated populations that are not swamped by gene flow from outbreaking populations (Chapuis et al., 2014). Previous single‐locus and microsatellite‐based studies on different locust species have found very shallow patterns of genetic structure at local/regional scales (e.g., Chapuis et al., 2011), no genetic differentiation between gregarious and solitary phase populations (Chapuis et al., 2008), higher levels of gene flow among outbreaking populations than among recession or nonoutbreak populations (Chapuis et al., 2009; Ibrahim, 2001; Ibrahim et al., 2000), and a little impact of recession periods on genetic diversity (Chapco & Litzenberger, 2004; Chapuis et al., 2014; Ibrahim et al., 2000). However, with the exception of the recent sequencing and annotation of the locust *Locusta migratoria* genome (Wang et al., 2014) and the identification of some genes linked to physiology, phase change, and dispersal capacity (Bakkali & Martín‐Blázquez, 2018; Ernst et al., 2015; Martín‐Blázquez, Chen, Kang, & Bakkali, 2017; Wang et al., 2014), high‐resolution genomic data have not been yet employed to determine fine‐spatial scale patterns of genetic structure, perform robust demographic inferences in outbreak and nonoutbreak populations, and assess the potential role of selective processes on shaping spatial patterns of genetic variation in these organisms of great economic importance (e.g., Crossley et al., 2017).

**Figure 1:**
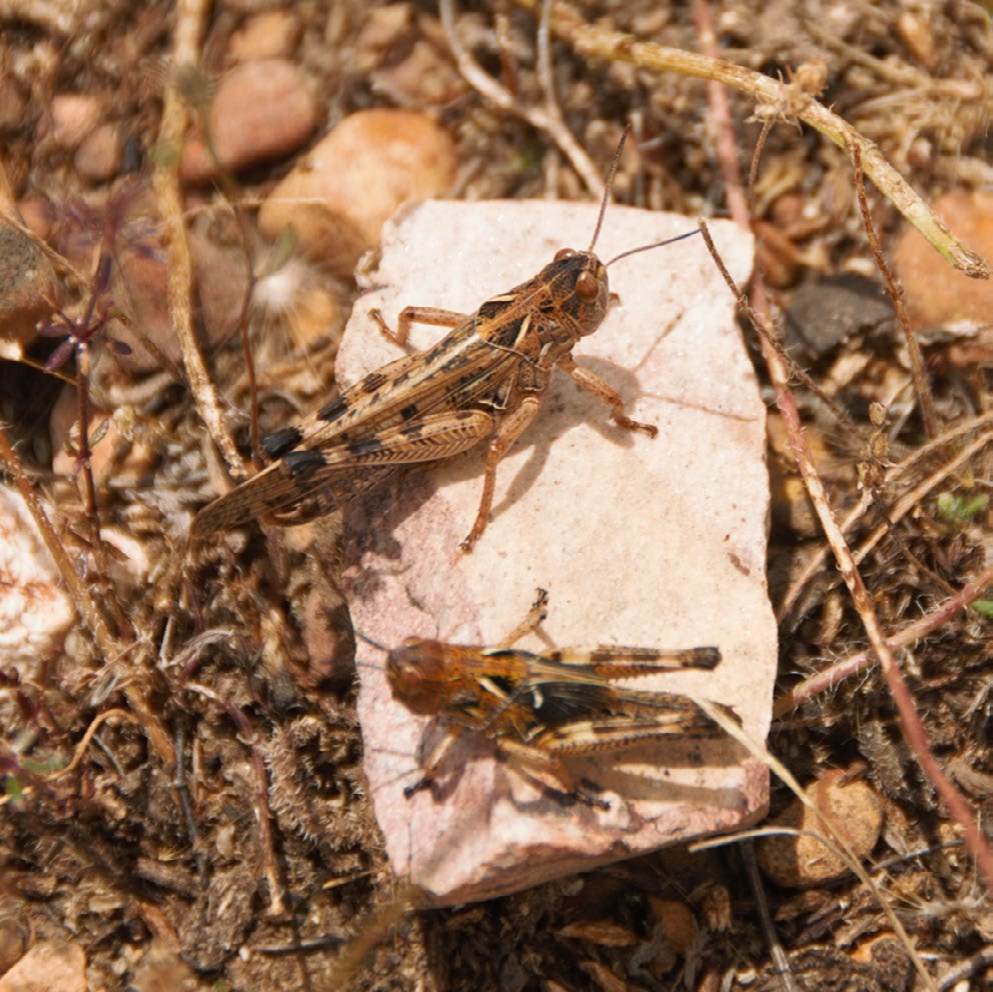
Adult male (top) and nymph (bottom) of Moroccan locust (*Dociostaurus maroccanus*) from Valle de Alcudia (Ciudad Real, Spain). Photograph by Pedro J. Cordero

The Moroccan locust, *Dociostaurus maroccanus* (Thunberg, 1815; Figure 1), is a xerophilous species distributed in most of the Western Palearctic, from the Canary Islands to south Kazakhstan (Cigliano, Braun, Eades, & Otte, 2019; Latchininsky, 1998). The species is characterized by its broad polyphagia, extreme voracity, enormous fecundity, extraordinarily fluctuating populating sizes, and high capability to migrate (del Cañizo & Moreno, 1949; el Ghadraoui, Petit, Picaud, & Yamani, 2002; Latchininsky, 1998; Uvarov, 1977). Its distribution is discontinuous and consists of fragmented nonoutbreaking populations in some areas and permanent foci of outbreak populations that cyclically become devastating agricultural pests (del Cañizo & Moreno, 1949; Latchininsky, 1998). It has been reported that Moroccan locusts can move distances of 70–100 km during their entire lifetime (rarely up to 200 km; Latchininsky, 1998), making possible the exchange of individuals between distant populations during swarming phases (Latchininsky, 1998). The species is considered a major agricultural pest of high economic importance, damaging pastures, and a wide variety of crops during outbreaks, which requires extensive control operations and chemical interventions with a tremendous cost year after year in affected countries (e.g., Arias‐Giralda, Jiménez‐Viñuelas, & Pérez‐Romero, 1997; Guerrero et al., 2019; Latchininsky, 2013).

Here, we focus on populations of the Moroccan locust from the Iberian Peninsula and the Canary Islands, which represent the west margin of the species’ distribution (Latchininsky, 2013). In the Iberian Peninsula, there are three main vast regions that are foci of population outbreaks and have suffered considerable damages to pastures and crops for centuries: Monegros (Aragón), La Serena (Extremadura) and Valle de Alcudia (Castilla‐La Mancha) (Alberola‐Roma, 2012; Arias‐Giralda, Morales‐Agacino, Cobos‐Suárez, Sopeña‐Mañas, & Martín‐Bernal, 1993). These regions are characterized by considerable cattle overgrazing, a factor that has been related to irruptive population growth in the Moroccan locust (Latchininsky, 1998; Louveaux, Mouhim, Roux, Gillon, & Barral, 1996). Besides, there are other regions from the Iberian Peninsula that represent historically outbreak areas that nowadays only sustain small populations or where the species has traditionally occurred at very low densities in isolated pockets of suitable habitat (Aragón, Coca‐Abia, Llorente, & Lobo, 2013; Latchininsky, 1998). The small size of some formerly outbreak populations has been hypothesized to be related with the expansion of agriculture and certain plowing techniques, the destruction and fragmentation of suitable breeding areas linked to land use changes, and the massive application of pesticides to control locusts’ populations (Latchininsky, 1998). On this respect, several Moroccan locust populations from outbreak areas have been considerably reduced by human intervention to the point that many of them have almost disappeared in the last decades (Aragón et al., 2013; Latchininsky, 1998). However, no study has been performed so far to understand the degree of genetic connectivity among populations of the Moroccan locust at regional scales, infer its past demographic history, or determine the potential role of local adaptation processes, information that might help to shed light on key aspects of the ecology, distribution, and evolutionary dynamics of this economically important species.

In this study, we use genomic data obtained via restriction site‐associated DNA sequencing (ddRADseq) to investigate the relative role of neutral versus selective processes on shaping genetic variation in the Moroccan locust, determine spatial patterns of genetic diversity and structure in solitary and outbreaking populations from the westernmost portion of the distribution of the species, and, ultimately, infer its past demographic history. First, we performed genome scans and environmental association analyses to identify putative loci under selection and evaluate the potential importance of local adaptation processes on shaping genetic differentiation at non‐neutral genomic regions. Second, we calculated different estimates of genetic diversity and performed a comprehensive suite of analyses of genetic structure to test the hypothesis of lower levels of genetic variation and increased genetic differentiation in solitary than outbreak populations (Chapuis et al., 2014). Finally, we used genomic data to infer the past demography of the studied populations. Specifically, we predict genomic signatures of recent demographic declines in solitary populations and formerly outbreaking populations that have undergone remarkable retreats during the last decades (Chapuis et al., 2014; Ibrahim et al., 2000) and, given the thermophilous character of the Moroccan locust, we also expect that the species has experienced historical bottlenecks during Pleistocene glacial periods and population expansions in interglacials (Meco et al., 2011).

## 2 MATERIALS AND METHODS

### 2.1 Population sampling

**Figure 2:**
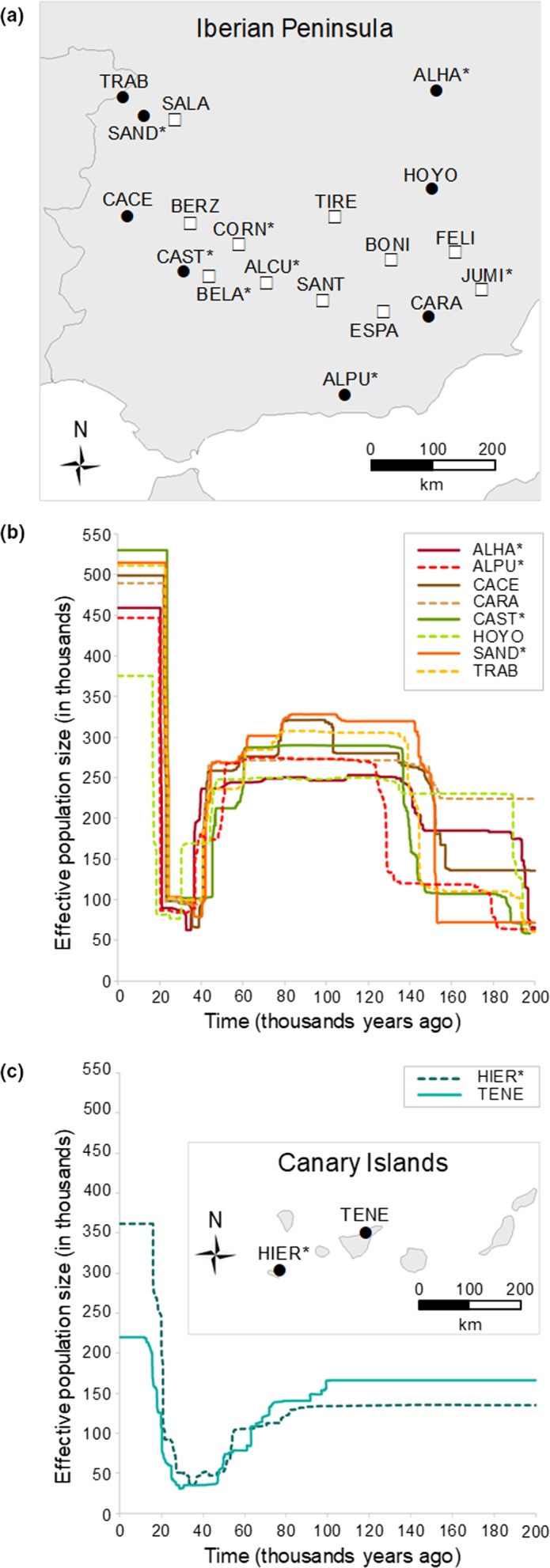
Geographic location of the studied populations of Moroccan locust in (a) the Iberian Peninsula and (c) the Canary Islands. Black dots indicate those populations analyzed with stairway plot (*n* = 8 individuals) and white squares the rest of the populations (*n* < 8 individuals). Panels (b) and (c) present the inferred demographic profiles for Iberian and Canarian populations, respectively. Lines show the median estimate of effective population size *(N*_e_) over time, assuming a mutation rate of 2.8 × 10^–9^ and 1‐year generation time. Populations with an asterisk indicate pest outbreaks during the sampling year. Population codes are described in Table 1.

Between May and July 2011–2016, we prospected adequate habitats for the Moroccan locust (*Dociostaurus maroccanus*) (i.e., grazed grasslands, natural sparse vegetation, arid or semidesert steppes, and abandoned agricultural fields; Latchininsky, 1998; Latchininsky, 2013) in the Iberian Peninsula and the Canary Islands. We sampled a total of 21 localities representative of both outbreak and nonoutbreak populations (Figure 2; Table 1), a status defined according with our own field observations during sampling (i.e., densities of more than ~20 individuals/m^2^ were considered as outbreak populations) and corroborated with information provided by regional government authorities implementing pest management programs. Information about the outbreak or nonoutbreak status of the different sampling populations is presented in Table 1. Adult individuals were collected via sweep netting in an area not higher than 500 m^2^ around each sampling locality. Fresh whole adult specimens were placed in vials with 2–5 ml ethanol 96% and stored at −20°C until needed for genomic analyses. For this study, we analyzed a total of 5–8 adult individuals per locality (Table 1).

**Table 1:** Geographical location of the studied populations of Moroccan locust, population codes, sampling year, number of individuals per population (*n*), and scores for the three environmental principal components used in LFMM analyses (PC1, PC2, and PC3). Populations with an asterisk indicate pest outbreaks during the sampling year. Average values of genetic statistics across neutral loci are presented for major allele frequency (*P*), observed (*H*_O_) and expected (*H*_E_) heterozygosity, nucleotide diversity (π), and the Wright's inbreeding coefficient (*F*_IS_) for all positions (polymorphic and nonpolymorphic)

Locality (Province)	Code	Year	*n*	Latitude	Longitude	PC1	PC2	PC3	*P*	*H*_O_	*H*_E_	π	*F*_IS_
Trabanca (Salamanca)	TRAB	2015	8	41.24275	−6.40222	−0.447	−0.283	1.282	0.9994	0.0007	0.0009	0.0010	0.0007
Sando (Salamanca)*	SAND	2015	8	40.96961	−6.10954	−0.573	−0.279	0.630	0.9994	0.0007	0.0009	0.0010	0.0007
Salamanca (Salamanca)	SALA	2015	5	40.93764	−5.66806	−0.608	−0.335	−0.726	0.9994	0.0007	0.0009	0.0010	0.0006
Alhama de Aragón (Zaragoza)*	ALHA	2015	8	41.34571	−1.92277	−1.613	−0.820	−0.939	0.9994	0.0007	0.0009	0.0010	0.0007
Los Llanos de Cáceres (Cáceres)	CACE	2015	8	39.53310	−6.32455	1.072	0.887	0.094	0.9994	0.0007	0.0009	0.0010	0.0008
Puerto de la Berzocana (Cáceres)	BERZ	2012	5	39.44998	−5.42609	−0.349	0.401	1.006	0.9994	0.0007	0.0008	0.0009	0.0005
Castuera (Cáceres)*	CAST	2015	8	38.74827	−5.54450	1.036	1.186	0.466	0.9994	0.0007	0.0009	0.0010	0.0007
Cañada del Hoyo (Cuenca)	HOYO	2015	8	39.93709	−1.99317	−1.787	−0.467	0.010	0.9994	0.0007	0.0009	0.0010	0.0007
Laguna de Tirez (Toledo)	TIRE	2015	7	39.54259	−3.35907	0.144	0.660	−1.063	0.9994	0.0007	0.0009	0.0010	0.0007
Raña Cornicabra (Ciudad Real)*	CORN	2011	5	39.13134	−4.73814	0.550	0.924	−0.471	0.9994	0.0007	0.0009	0.0010	0.0005
Valle de Alcudia (Ciudad Real)*	ALCU	2015	7	38.58958	−4.35354	0.560	1.059	0.502	0.9994	0.0008	0.0009	0.0010	0.0006
Belalcázar (Córdoba)*	BELA	2015	5	38.67942	−5.16172	1.125	1.311	0.342	0.9994	0.0008	0.0009	0.0010	0.0005
El Bonillo (Albacete)	BONI	2015	7	38.93758	−2.56544	−0.528	0.209	−0.507	0.9994	0.0007	0.0009	0.0010	0.0008
La Felipa (Albacete)	FELI	2015	5	39.03552	−1.64832	−0.299	−0.185	−1.642	0.9994	0.0007	0.0009	0.0010	0.0005
Santa Elena (Jaén)	SANT	2015	5	38.33753	−3.51923	0.594	0.935	0.098	0.9994	0.0008	0.0009	0.0010	0.0005
Santiago de la Espada (Jaén)	ESPA	2011	5	38.18128	−2.66677	−1.238	−0.058	0.920	0.9994	0.0007	0.0009	0.0010	0.0005
Jumilla (Murcia)*	JUMI	2015	5	38.49016	−1.24497	0.228	−0.237	−1.815	0.9994	0.0007	0.0009	0.0010	0.0005
Caravaca de la Cruz (Murcia)*	CARA	2015	8	38.10283	−2.01999	−0.208	−0.053	−0.895	0.9994	0.0007	0.0009	0.0010	0.0007
Alpujarra (Granada)*	ALPU	2015	8	36.96846	−3.21425	−0.986	−0.101	1.811	0.9994	0.0007	0.0009	0.0010	0.0007
Tenerife (Santa Cruz de Tenerife)	TENE	2016	8	28.41569	−16.40991	1.343	−2.257	1.449	0.9995	0.0006	0.0007	0.0008	0.0004
El Hierro (Santa Cruz de Tenerife)*	HIER	2016	8	27.77303	−17.95796	1.984	−2.497	−0.555	0.9995	0.0006	0.0007	0.0008	0.0004

### 2.2 Genomic library preparation

We used NucleoSpin Tissue kits (Macherey‐Nagel, Durën, Germany) to extract and purify genomic DNA from the hind femur of each individual. Genomic DNA from 141 individuals of the 21 sampling localities (Table 1) was processed into three genomic libraries (47 individuals per library) using the double‐digestion restriction site‐associated DNA sequencing procedure (ddRADseq) described in Peterson, Weber, Kay, Fisher, and Hoekstra (2012). In brief, DNA was doubly digested with the restriction enzymes *Mse*I and *Eco*R1 (New England Biolabs, Ipswich, MA, USA) and Illumina adaptors including unique 7‐bp barcodes were ligated to the digested fragments. Ligation products from individuals assigned to each library were pooled, size‐selected between 475 and 580 bp with a Pippin Prep (Sage Science, Beverly, MA, USA) machine, and amplified by PCR with 12 cycles using the iProof^TM^ High‐Fidelity DNA Polymerase (BIO‐RAD, Hercules, CA, USA). Each library was sequenced in single‐read 150‐bp lane on an Illumina HiSeq2500 platform at The Centre for Applied Genomics (SickKids, Toronto, ON, Canada).

### 2.3 Genomic data analyses

We used the different programs distributed as part of the stacks v. 1.35 pipeline (*process_radtags*, *ustacks*, *cstacks*, *sstacks*, and *populations*) to assemble our sequences into de novo loci and call genotypes (Catchen, Amores, Hohenlohe, Cresko, & Postlethwait, 2011; Catchen, Hohenlohe, Bassham, Amores, & Cresko, 2013a; Hohenlohe et al., 2010). For the different filtering and assembling steps with stacks, we used the default parameters recommended by the authors (Catchen et al., 2011; Catchen, Hohenlohe, et al., 2013; Hohenlohe et al., 2010). Reads were demultiplexed and filtered for overall quality using the program *process_radtags*, retaining reads with a Phred score > 10 (using a sliding window of 15%), no adaptor contamination, and that had an unambiguous barcode and restriction cut site. Raw reads were screened for quality with fastqc v. 0.11.5 (http://www.bioinformatics.babraham.ac.uk/projects/fastqc), and all sequences were trimmed to 129‐bp using seqtk (https://github.com/lh3/seqtk) in order to remove low‐quality reads near the 3´ ends. Filtered reads of each individual were assembled de novo into putative loci with the *ustacks* program. The minimum stack depth (*m*) was set to three, and we allowed a maximum distance of two nucleotide mismatches (*M*) to group reads into a “stack.” We used the “removal” (*r*) and “deleveraging” (*d*) algorithms to eliminate highly repetitive stacks and resolve over‐merged loci, respectively. Single nucleotide polymorphisms (SNPs) were identified at each locus, and genotypes were called using a multinomial‐based likelihood model that accounts for sequencing errors, with the upper bound of the error rate (*ε*) set to 0.2 (Catchen et al., 2011; Catchen, Hohenlohe, et al., 2013; Hohenlohe et al., 2010). A catalogue of loci was built using the *cstacks* program, with loci recognized as homologous across individuals if the number of nucleotide mismatches between consensus sequences (*n*) was ≤2. Each individual data were matched against this catalogue using *sstacks* program, and output files were exported in different formats for subsequent analyses using the program *populations*. For all downstream analyses, we exported only the first SNP per RAD locus (option *write_single_snp*) and retained loci with a minimum stack depth ≥ 5 (*m* = 5), that were sequenced in at least 50% of the individuals of each population (parameter *r* = 0.5), represented in ~66% of populations, and with a minimum minor allele frequency (MAF) ≥ 0.01 to reduce the number of false polymorphic loci due to sequencing errors. As demonstrated in previous studies, the choice of different filtering thresholds affecting the proportion of missing data (*p* and *r* in program *populations*) had little impact on the obtained inferences (e.g., González‐Serna, Cordero, & Ortego, 2018; González‐Serna, Cordero, & Ortego, 2019; Ortego, Gugger, & Sork, 2018). The resulting files were used for subsequent analyses or converted into other formats using the program pgdspider v.2.1.0.3 (Lischer & Excoffier, 2012).

### 2.4 Outlier loci detection and environmental association analyses

In a first step, we screened for loci not conforming to neutral expectations using two outlier detection approaches: the coalescent‐based *FDIST* method from arlequin (Excoffier & Lischer, 2010) and the Bayesian approach implemented in bayescan v.2.1 (Foll & Gaggiotti, 2008). The *FDIST* method in arlequin was run in two different ways, considering both the nonhierarchical (Beaumont & Nichols, 1996) and hierarchical (Excoffier, Hofer, & Foll, 2009) island models. The nonhierarchical island model was run using 200,000 simulations, 100 demes, and expected heterozygosity ranging from 0 to 1. The hierarchical island model was run grouping populations according to their geographical origin and the results obtained from genetic structure analyses (i.e., Iberian versus Canarian populations; see Results section), using the same settings as the nonhierarchical model, and considering three simulated groups (i.e., the number of defined population groups plus one, as recommended in Excoffier and Lischer (2010). *P*‐values were corrected for multiple testing using the *p.adjust* function in r (R Core Team, 2018) and loci significantly outside the neutral distribution at a false discovery rate (FDR) of 5% (i.e., *q* < 0.05) were considered as outliers. bayescan analyses were run under default settings (thinning interval size of 10; 20 pilot runs of 5,000 iterations; burn‐in length of 50,000 iterations), except for an increase of outputted iterations to 10,000. We used 10 (default) prior odds and adopted the same FDR to identify candidate loci putatively under selection as in arlequin analyses (FDR of 5%, *q* < 0.05). bayescan and arlequin analyses were run independently for two different genomic datasets, one considering all populations and another one only considering populations from the Iberian Peninsula (e.g., Guo et al., 2016).

In a second step, we performed environmental association analyses (EAA) using Latent Factor Mixed Models (LFMM) implemented in the r v.3.3.3 (R Core Team, 2018) package lea (Frichot & François, 2015). This approach uses a stochastic Monte Carlo Markov Chain algorithm to test for associations between environmental/ecological variables and allele frequencies while simultaneously controlling for background levels of population structure (Frichot, Schoville, Bouchard, & François, 2013). As environmental information, we used the 19 bioclimatic variables from the worldclim dataset interpolated to 30‐arcsec resolution (~1 km^2^ cell size) (Hijmans, Cameron, Parra, Jones, & Jarvis, 2005). These variables summarize information about temperature and precipitation and have been commonly used in exploratory analyses aimed to test the broad hypothesis of environment‐driven selection (e.g., François, Martins, Caye, & Schoville, 2016; Frichot & François, 2015; de Kort et al., 2014; Rellstab et al., 2015). We extracted the values for these variables from all adjacent cells around each sampling location (i.e., ~9 km^2^) using bilinear interpolations as implemented in arcgis 10.3 (ESRI, Redlands, CA, USA). To summarize and reduce strong redundancy among the 19 bioclimatic variables, we ran a principal component analysis (PCA) on all of them (e.g., François et al., 2016; Frichot & François, 2015; de Kort et al., 2014). The first three principal components (PCs) cumulatively accounted for 94.82% of the variance (PC1: 38.58%; PC2: 33.77%; PC3: 22.47%) and were retained for LFMM analyses (Table A1). The contribution of bioclimatic variables to each axis (i.e., the factor loadings for each PC) is presented in Table 1. The MCMC algorithm was implemented for each of the three PCs (i.e., PC1, PC2, and PC3), using 10,000 iterations, 5,000 as burning period, and 5 independent replicates of the analysis. As indicated above for bayescan and nonhierarchical arlequin analyses, LFMM analyses were run independently for all populations and only considering Iberian populations. The number of latent factors included in the model as a covariate to control for demographic history were defined on the basis of structure analyses (Pritchard, Stephens, & Donnelly, 2000; see Results section) and sparse non‐negative matrix factorization (*snmf*) analyses implemented in the r package lea (Frichot, Mathieu, Trouillon, Bouchard, & Francois, 2014). We set the number of latent factors (*K*) at *K* = 2 for analyses including all populations and *K* = 1 for analyses focused on Iberian populations. The *z*‐scores over the five replicates were combined and recalibrated using the genomic inflation factor (λ) (Frichot & François, 2015). Finally, we performed a FDR adjustment to control for multiple tests (FDR of 5%, *q* < 0.05) and identify putative loci under environmental selection for each of the three PCs summarizing bioclimatic variation.

### 2.5 Genetic structure

We employed three complementary approaches to exhaustively explore spatial patterns of genetic structure in our study system, including (a) principal component analyses (PCA) (Jombart, 2008); (b) classic structure analyses considering and not considering prior population information (Hubisz, Falush, Stephens, & Pritchard, 2009; Pritchard et al., 2000); and (c) the recently developed spatial method implemented in the r program construct (Bradburd, Coop, & Ralph, 2018). In all cases, genetic structure was analyzed only considering putatively neutral loci. To this end, outlier loci detected by arlequin and bayescan and SNPs identified by LFMM analyses as being putatively under environment‐driven selection (a total of 9,346 loci; see Section 3) were conservatively excluded to create datasets only containing neutral loci (e.g., Brauer, Hammer, & Beheregaray, 2016; Ortego et al., 2018). This yielded neutral datasets of 40,179 SNPs for all populations, 42,114 SNPs for Iberian populations, and 33,998 SNPs for Canarian populations.

#### 2.5.1 Principal component analyses (PCA)

In order to visualize the major axes of population genetic differentiation, we performed individual‐based principal component analyses (PCA) using the r package adegenet (Jombart, 2008). Before running the PCA, we scaled and centered allele frequencies and replaced missing data with mean allele frequencies using the *scalegen* function as recommended by Jombart (2008). PCAs were run using all neutral SNPs for the two main datasets (all populations and Iberian Peninsula).

#### 2.5.2 structure analyses

We inferred genetic structure at neutral loci using the Bayesian Markov chain Monte Carlo clustering method implemented in the program structure v.2.3.3 (Falush, Stephens, & Pritchard, 2003; Hubisz et al., 2009; Pritchard et al., 2000). We conducted structure analyses hierarchically, initially analyzing data from all populations jointly and, subsequently, running independent analyses for subsets of populations assigned to the same genetic cluster in the previous hierarchical level analysis (e.g., Coulon et al., 2008; González‐Serna et al., 2019). To make analyses computationally tractable, we ran structure using a single random subset of 10,000 unlinked neutral SNPs. According to previous studies, this number of loci is an order of magnitude higher than that necessary to obtain robust and reproducible results in structure (e.g., Catchen, Bassham, et al., 2013:1,000 loci; González‐Serna et al., 2019:1,250 loci). We ran structure using 200,000 MCMC iterations after a burn‐in step of 100,000 iterations, assuming correlated allele frequencies and admixture, and both considering and not considering prior population information (Hubisz et al., 2009). We performed 15 independent runs for each value of *K*. In order to ensure analysis convergence, we only retained the ten runs having the highest likelihood for each value of *K* (e.g., Yannic et al., 2018) and checked that all retained replicates reached a similar solution in terms of individual's probabilities of assignment to each genetic cluster (*q*‐values; Gilbert et al., 2012). As recommended, we used two statistics to identify the most likely number of genetic clusters (*K*) (Gilbert et al., 2012; Janes et al., 2017): log probabilities of Pr(X|*K*) (Pritchard et al., 2000) and the Δ*K* method (Evanno, Regnaut, & Goudet, 2005). These statistics were calculated as implemented in structure harvester (Earl & vonHoldt, 2012). Finally, we used clumpp v. 1.1.2 and the Greedy algorithm to align multiple runs of structure for the same *K* value (Jakobsson & Rosenberg, 2007) and distruct v. 1.1 (Rosenberg, 2004) to visualize as bar plots the individual's probabilities of membership to each inferred genetic cluster.

#### 2.5.3 construct analyses

We used the spatial model implemented in r package construct v. 1.0.3 to infer patterns of genetic structure across the Iberian populations and determine whether genetic differentiation is a consequence of continuous (i.e., isolation‐by‐distance) or discrete (e.g., separation by geographic barriers, etc.) processes (Bradburd et al., 2018). Given that this approach is highly sensitive to missing data, we analyzed a database with no missing data (2,904 unlinked SNPs) as recommended by the authors (Bradburd et al., 2018). We ran construct analyses with 5,000 iterations and visually checked for convergence using trace plots (Bradburd et al., 2018). We used a fivefold cross‐validation approach to examine predictive accuracy across the range of tested *K* values and determine the best‐fit number of genetic clusters (Bradburd et al., 2018). As done for structure, we plotted individual coancestry coefficients for the different *K* values using distruct.

### 2.6 Geographical and environmental drivers of genetic differentiation

We tested for the presence of isolation‐by‐distance (IBD) and/or isolation‐by‐environment (IBE) patterns of genetic structure by analyzing the association between genetic differentiation (*F*_ST_) and geographic and environmental distances among Iberian populations (Sexton, Hangartner, & Hoffmann, 2014; Shafer & Wolf, 2013; Wang, 2013). Genetic differentiation (*F*_ST_) between all pairs of populations was calculated for the subset of neutral loci (see previous section) using the program *populations* from stacks (Table A2). Geographic distance between each pair of Iberian populations was calculated using geographic distance matrix generator v.1.2.3 (Ersts, 2018). Environmental distances were calculated for each PC (PC1, PC2, and PC3) obtained from a PCA on the 19 bioclimatic variables (see LFMM analyses above for details on PCA) using the “*dist*” function in r 3.3.3 (R Core Team, 2018). Genetic differentiation [*F*_ST_/(1 − *F*_ST_)] was tested against matrices of geographical (log10 transformed) and environmental distances using multiple matrix regressions with randomization (MMRR) as implemented in the “*mmrr*” function (Wang, 2013) in r 3.3.3 (R Core Team, 2018). Given that geographical/environmental distances are only expected to have positive effect on the degree of genetic differentiation between populations, we used one‐tailed hypothesis tests for making statistical decisions regarding the null hypothesis of no effect of independent variables on genetic differentiation (Ruxton & Neuhäuser, 2010; e.g., Berkman, Nielsen, Roy, & Heist, 2013; Yannic et al., 2018). We selected final models following a backward procedure, initially fitting all explanatory terms and progressively removing nonsignificant variables until all retained variables were significant. The significance of the variables excluded from the model was tested again until no additional term reached significance (e.g., Ortego, Aguirre, Noguerales, & Cordero, 2015a).

### 2.7 Genetic diversity and past demographic history

We only employed neutral loci for calculating genetic diversity statistics and performing demographic inference analyses (Luikart et al., 2003). We used the program *populations* from stacks to calculate some genetic statistics, including nucleotide diversity (π), observed (*H*_O_) and expected (*H*_E_) heterozygosity, major allele frequency (*P*), and the Wright's inbreeding coefficient (*F*_IS_) (Catchen, Hohenlohe, et al., 2013). Standardized multilocus heterozygosity (sMLH) was calculated for each individual using the r package inbreedr (Stoffel et al., 2016). sMLH is an individual‐based metric defined as the total number of heterozygous loci in an individual divided by the sum of average observed heterozygosities in the population, over the subset of loci successfully typed in the focal individual (Coltman, Pilkington, Smith, & Pemberton, 1999).

We used stairway plot (Liu & Fu, 2015) to reconstruct the demographic history of the studied populations, a novel model‐flexible method based on the site frequency spectrum (SFS) that does not require whole‐genome sequence data or reference genome information to infer changes in effective population size (*N*_e_) over time. These analyses were restricted to populations with eight genotyped individuals (see Figure 2 and Table 1), as the calculation of the SFS requires a downsampling procedure to remove missing data. These populations are representative of the distribution of the species across the study area (see Figure 2a). To compute the SFS for each population, we ran the program *populations* from stacks (Catchen, Hohenlohe, et al., 2013) in order to export the first SNP per RAD locus and retain loci with a minimum stack depth ≥ 5 (*m* = 5) and that were represented in at least 50% of the individuals of the focal population (*r* = 0.5). To remove all missing data for the calculation of the SFS and minimize errors with allele frequency estimates, each population was down‐sampled to 6 individuals using a custom Python script written by Qixin He and available on Dryad (Papadopoulou & Knowles, 2015). We ran stairway plot for each population fitting a flexible multi‐epoch demographic model, assuming the mutation rate per site per generation of 2.8 × 10^–9^ estimated for *Drosophila melanogaster* (Keightley, Ness, Halligan, & Haddrill, 2014), a one‐year generation time (Latchininsky, 1998), four different number of random breakpoints [(nseq‐2)/4, (nseq‐2)/2, (nseq‐2)*3/4, and nseq‐2], and 200 bootstrap replicates to estimate 95% confidence intervals.

## 3 RESULTS

### 3.1 Genomic data analyses

Illumina sequencing of ddRAD libraries generated > 358 millions of reads in total after first quality filtering using the program *process_radtags*. The number of reads per individual before and after different quality filtering steps is shown in Figure A1. Only one sample from population BONI was excluded for subsequent analyses due to low number of reads (Figure A1). The dataset obtained with stacks for all populations contained a total of 49,373 unlinked SNPs.

### 3.2 Outlier loci detection and environmental association analyses

**Figure 3:**
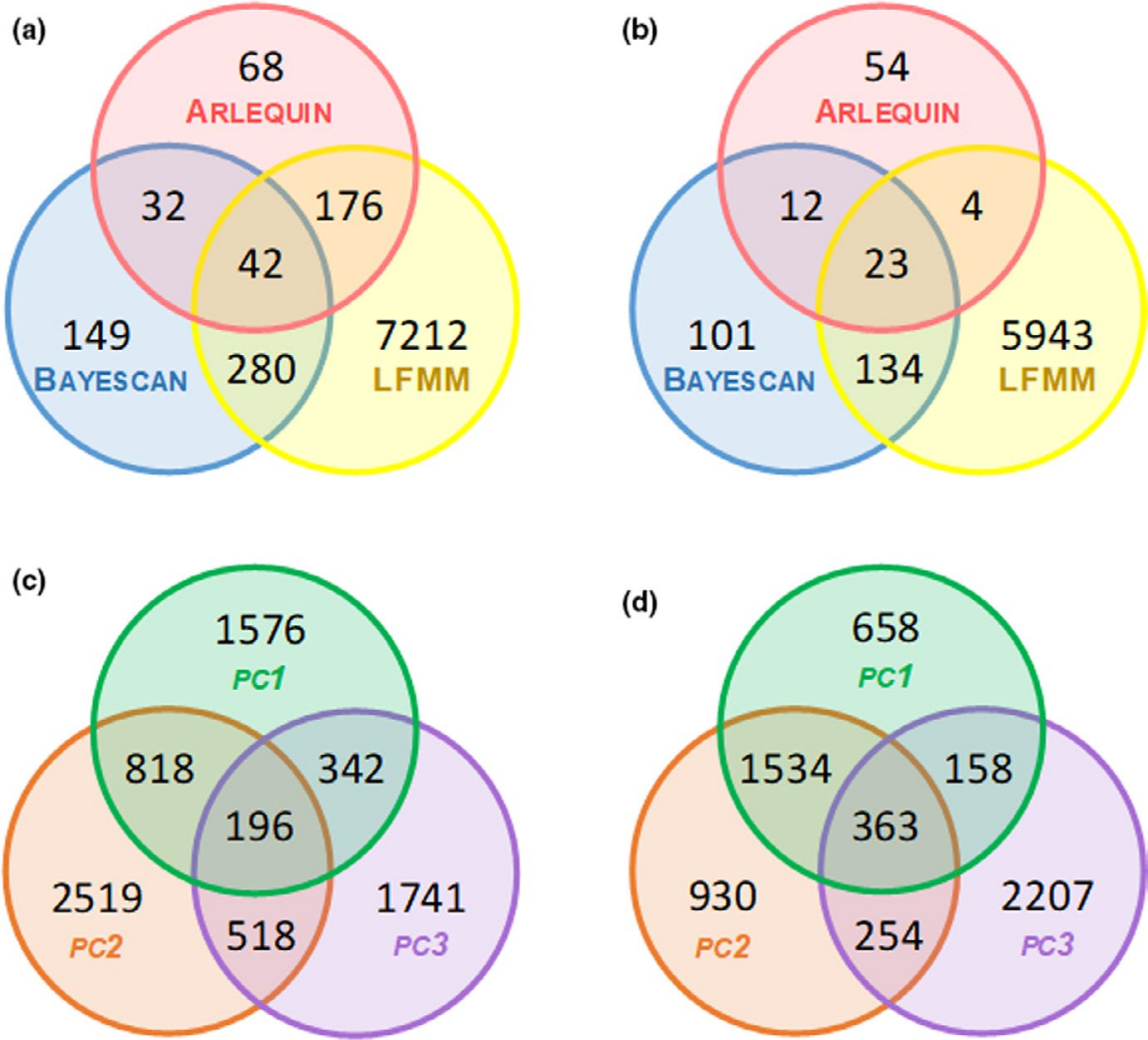
Venn diagrams showing the overlap of number of loci identified as being putatively under selection by arlequin (*FDIST* method) (red circles) and bayescan (blue circles) and presenting environmental associations according to LFMM (yellow circles) for analyses based on (a) all populations and (b) restricted to populations from the Iberian Peninsula. Panels (c–d) show the number of loci presenting associations with environmental PC1 (green circles), PC2 (orange circles), and PC3 (purple circles) according to LFMM analyses based on (c) all populations and (d) restricted to populations from the Iberian Peninsula.

For the dataset including all populations, we identified 318 (0.64%) outlier loci using arlequin (*FDIST* method) and 503 (1.02%) using bayescan (Figures 3a,b). When the analyses were restricted to Iberian populations, we identified 93 (0.18%) outlier loci using arlequin and 270 (0.52%) using Bayescan (Figure 3a,b). Several SNPs were commonly identified as outliers by both arlequin and bayescan analyses (all populations: 74 SNPs, 0.15%, Figure 3a; Iberian populations: 35 SNPs, 0.07%, Figure 3b). Most outlier loci were identified as being putatively under divergent selection in analyses based on both the datasets including all populations (arlequin: *n* = 275, 86.47%; bayescan: *n* = 467, 92.84%) and the one restricted to Iberian populations (arlequin: *n* = 76, 81.72%; bayescan: *n* = 269, 99.63%). Environmental association analyses in LFMM showed that a high number of loci were significantly associated with environmental variation (Figure 3c,d). For the dataset including all populations, LFMM detected 7,710 unique loci (15.62%) associated with at least one PC of environmental variation (PC1: 2,932 loci; PC2: 4,051 loci; PC3: 2,797 loci; Figure A2) and 196 of them (0.40%) showed significant associations with the three PCs (Figure 3c). Similarly, when the analyses were restricted to Iberian populations, LFMM detected 6,104 unique loci (11.87%) associated with at least one PC of environmental variation (PC1: 2,713 loci; PC2: 3,081 loci; PC3: 2,982 loci; Figure A2) of which 363 (0.71%) were shared across all PCs (Figure 3d). Only 42 loci (0.08%) for analyses based on all populations (Figure 3a) and 23 loci (0.04%) for analyses focused on Iberian populations (Figure 3b) showed associations with environmental variation in LFMM and were also identified as *F*_ST_ outliers by arlequin and bayescan analyses.

### 3.3 Genetic structure

**Figure 4:**
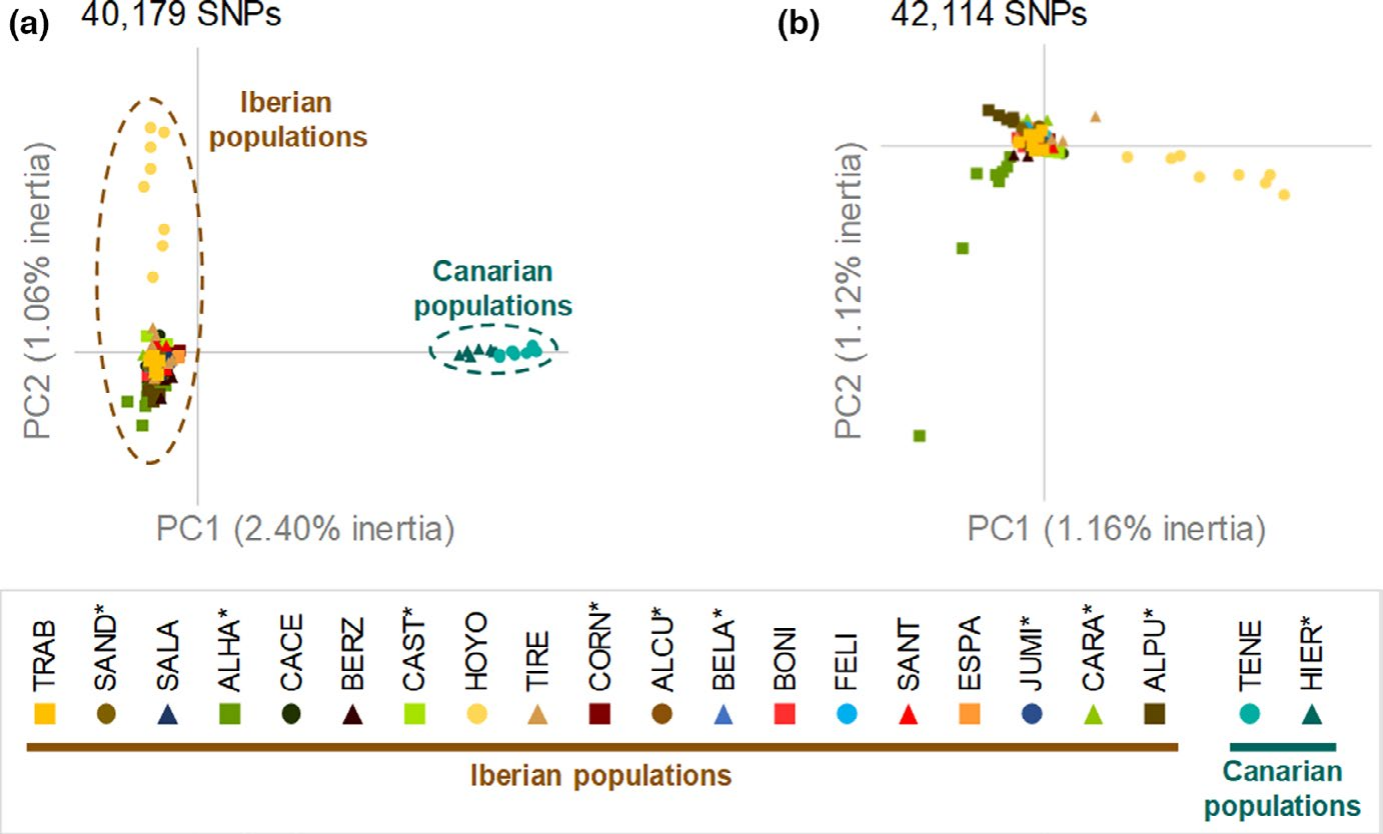
Principal component analyses (PCA) of genetic variation for populations of Moroccan locust for (a) all populations and (b) only considering populations from the Iberian Peninsula. Dashed brownish and greenish ellipses encircle Iberian and Canarian populations, respectively. The number of loci used in the different analyses is indicated in each panel. Populations with an asterisk indicate pest outbreaks during the sampling year. Population codes are described in Table 1

**Figure 5:**
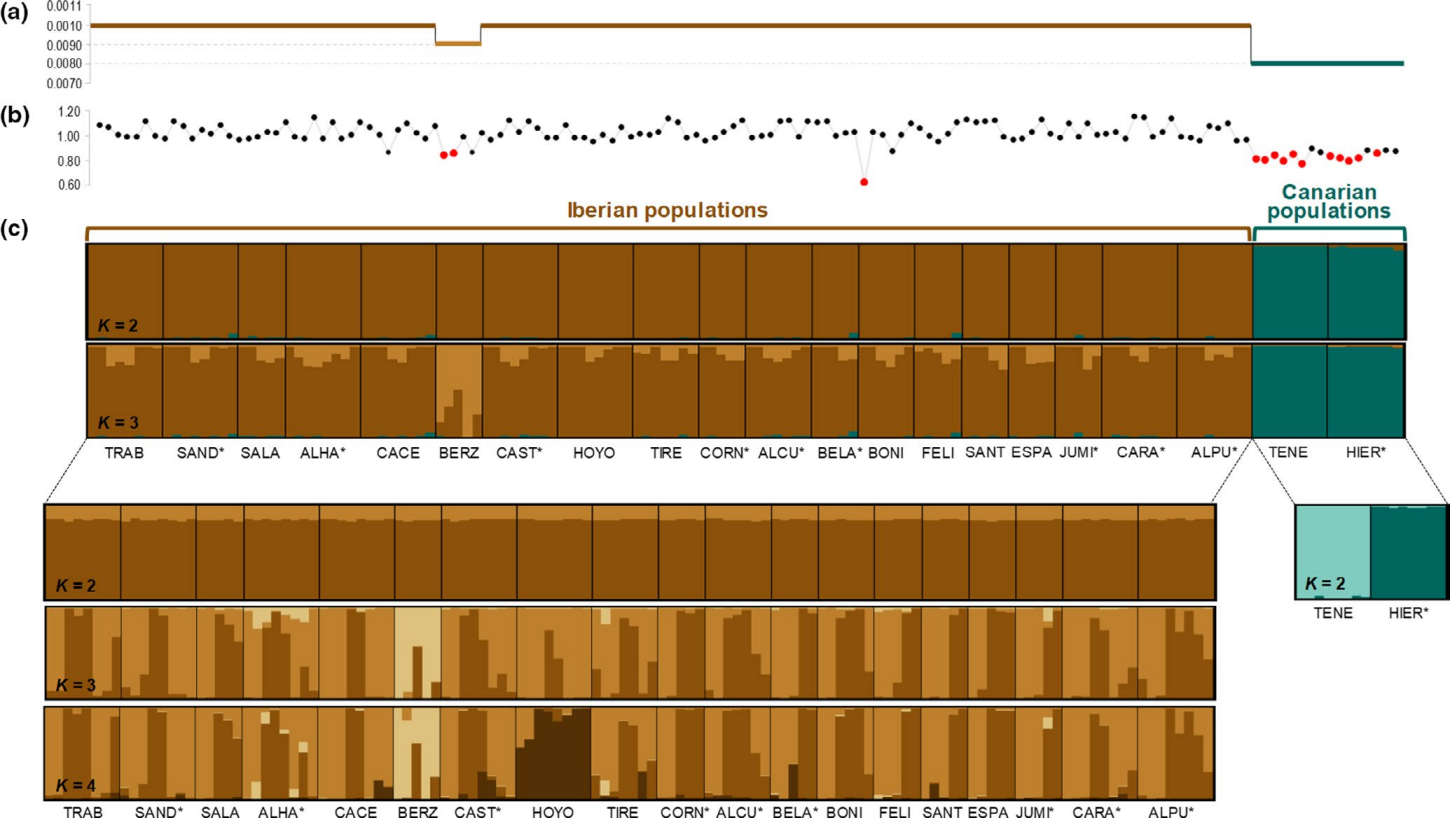
(a–b) Genetic diversity and (c) results of structure analyses not considering prior population information for the studied populations of Moroccan locust. Panel (a) shows nucleotide diversity (π) for each population calculated in stacks for all positions (polymorphic and nonpolymorphic). Panel (b) shows standardized multilocus heterozygosity (sMLH) of each individual, with values below the lowest 10th percentile shown in red. (c) Bar plots of structure analyses show the individual's probabilities of membership to each inferred genetic cluster for different *K* values. Each individual is represented by a vertical bar, which is partitioned into *k* colored segments showing the individual's probability of belonging to the cluster with that color. Thin vertical black lines separate individuals from different populations. structure analyses were performed at different hierarchical levels, analyzing all populations together and Iberian (brownish) and Canarian (greenish) populations separately. Populations with an asterisk indicate pest outbreaks during the sampling year. Population codes are described in Table 1.

The PCA based on all populations showed a clear separation of Iberian and Canarian populations (Figure 4a). The PCA for the subset of Iberian populations showed that HOYO was the most differentiated population and some individuals from two other populations (ALHA and ALPU) also tended to stand out from the rest (Figure 4b). These results are in good agreement with those obtained from Bayesian clustering analyses. structure analyses for all populations and not considering prior population information identified *K* = 2 as the most likely clustering solution according to both the Δ*K* criterion and log probabilities of the data [LnPr (X|*K*)] (Figure A3a). These two clusters showed a very low degree of genetic admixture and split Canarian and Iberian populations (Figure 5c). Remarkably, genetic drift after divergence (parameter *F* in structure; Pritchard et al., 2000) for the cluster corresponding to the Canary Islands (*F*‐value = 0.419) was more than threefold the estimated for Iberian populations (*F*‐value = 0.121). structure analyses for *K* = 3 revealed further genetic structure and showed that one population from the Iberian Peninsula (BERZ) split from the rest of the mainland populations, albeit with a high degree of genetic admixture with the other Iberian populations (~10%–20%; Figure 5c). structure analyses restricted to Iberian populations identified that the most likely number of clusters was *K* = 2 according to the Δ*K* criterion, but LnPr (X|*K*) steadily increased up to *K* = 4 (Figure A3c). For *K* = 2, all the Iberian populations and individuals presented the same proportion of ancestry to the two inferred clusters (~20/80), indicating that they represent ghost clusters with no biological significance (see Chen, Durand, Forbes, & François, 2007; Guillot, Estoup, Mortier, & Cosson, 2005; Tonzo, Papadopoulou, & Ortego, 2019). However, structure analyses for *K* = 3 and *K* = 4 showed that BERZ and HOYO were assigned to two different genetic groups albeit with some degree of genetic admixture with the rest of Iberian populations (Figure 5c). The genetic clusters corresponding to these two populations had much higher estimates of genetic drift after divergence (BERZ: *F*‐value = 0.263; HOYO: *F*‐value = 0.084) than the one representing the rest of Iberian populations (*F*‐value = 0.021). Finally, structure analyses restricted to populations from the Canary Islands showed *K* = 2 as the most likely clustering solution according to both the Δ*K* criterion and LnPr (X|*K*) (Figure A3e). These two clusters separated HIER and TENE populations, which showed a very low degree of genetic admixture (Figure 5c). structure analyses ran considering prior population information (Hubisz et al., 2009) yielded qualitatively similar results, albeit in this case BERZ and HOYO presented a very small probability of assignment to their specific clusters in analyses focused on Iberian populations for *K* = 3 and *K* = 4 (see Figures A3 and A4). Finally, spatial analyses in construct focused on Iberian populations also showed strong admixture and no clear pattern of genetic structure. The predictive accuracy of construct analyses sharply increased from *K* = 1 to *K* = 2 (Figure A5a). However, layers (i.e., genetic clusters) beyond *K* = 1 contributed relatively little to total covariance (Figure A5b). This was particularly remarkable for *K* = 2, in which the second layer contributed less than 3% to total covariance (Figure A5b). Accordingly, the inferred genetic clusters for *K* > 1 presented high genetic admixture with little geographical congruence and only BERZ for *K* = 2–3 and ALHA for *K* = 3 tended to be assigned to different genetic clusters (Figure A6).

### 3.4 Geographical and environmental drivers of genetic differentiation

Multiple matrix regression with randomization (MMRR) analyses showed that genetic differentiation [*F*_ST_/(1 − *F*_ST_)] among Iberian populations was not significantly associated with geographical or environmental (PC1, PC2, and PC3) distances (Table 2).

**Table 2:** Multiple matrix regression with randomization (MMRR) for genetic differentiation at neutral loci [*F*_ST_/(1 − *F*_ST_)] in relation with geographical (log10 transformed) and environmental distances between each pair of Iberian populations. Environmental distances (PC1, PC2, and PC3) were estimated from a principal component analysis (PCA) on the 19 bioclimatic variables from the worldclim dataset. *β*: regression coefficient; *t*: *t*‐statistic; *r* ^2^: coefficient of determination; *p*: one‐tailed significance level

	*β*	*t*	*r* ^2^	*p*
Geographic distance (log10)	−.666	−2.948	.049	.980
Environmental PC1	−.103	−2.081	.025	.853
Environmental PC2	−.080	−1.619	.015	.863
Environmental PC3	.025	0.492	.001	.397

### 3.5 Genetic diversity and past demographic history

Population genetic statistics (*P*, *H*_O_, *H*_E_, π, *F*_IS_) calculated in stacks for all positions (polymorphic and nonpolymorphic) are presented in Table 1. Population genetic diversity (π) and standardized multilocus heterozygosity (sMLH) for each individual are shown in Figure 5a. All estimates of population genetic diversity were significantly intercorrelated (*H*_O_ – *H*_E_: *r* = .681, *p* < .001; *H*_O_ – π: *r* = .681, *p* < .001; *H*_E_ – π: *r* = .99, *p* < .001; Table 1). Estimates of genetic diversity (*H*_O_: *r* = .99, *p* < .001; *H*_E_: *r* = .99, *p* < .001; π: *r* = .95, *p* < .001) and differentiation (*F*_ST_: *r* = .99, *p* < .001; see previous section) obtained from a subset of five individuals randomly sampled from populations with eight genotyped individuals were highly correlated with those estimated based on the full datasets. This indicates that a relatively small number of genotyped individuals per population (i.e., as few as five individuals, our minimum sample size; Table 1) provides reliable estimates of genetic diversity and differentiation (see also Ortego, Gugger, & Sork, 2015b). All estimates of population genetic diversity were significantly lower in Canarian than in Iberian populations (one‐way anovas; all *P*s < 0.001; Table 1) but did not significantly differ between outbreak and nonoutbreak populations (one‐way ANOVAs; all *P*s > 0.2; Table 1). stairway plot analyses showed no clear demographic differences between outbreak and nonoutbreak populations, so we grouped the demographic profiles inferred for the different populations according to the two main geographical regions: Iberian Peninsula and Canary Islands (Figure 2). All Iberian populations showed an ancestral population increase around 150 ka BP, a severe demographic bottleneck around the last glacial maximum (LGM; ~21 ka BP) with a *N*_e_ reduction by ~80% (from ~250,000 to ~50,000 diploid individuals), and an abrupt expansion at the onset of the Holocene to reach contemporary population sizes of around ~450,000 individuals (Figure 2b; Figure A7). The two Canarian populations presented a similar demographic profile than the Iberian ones although with a relatively less pronounced decline in *N*_e_ during the last glacial period (Figure 2c). Canarian populations showed a long period of stability in the past (200–100 ka BP) followed by a demographic bottleneck during the last glacial period (100–21 ka BP) and a subsequent expansion at the onset of the Holocene to reach contemporary effective population sizes of 220,000–350,000 diploid individuals (Figure 2c; Figure A7).

## 4 DISCUSSION

We employed a large single‐nucleotide polymorphism dataset (~50,000 loci) to infer historical demographic trends and understand the neutral and selective processes shaping spatial patterns of genomic variation in the Moroccan locust, a grasshopper that has traditionally emerged as an devastating pest in overgrazed areas and caused extensive agricultural damage in Spain (e.g., Buj‐Buj, 1992) and many other areas across its ample distribution range (Latchininsky, 1998). Our analyses, focused on the Iberian Peninsula and remote populations from the Canary Islands, indicated that the species is characterized by widespread gene flow and low levels of genetic differentiation at regional scales. They also showed little differences in past demography and levels of genetic diversity and differentiation between outbreak and nonoutbreak populations. Finally, genome scans revealed that a small fraction (~0.2%–1.0%) of the sequenced loci are putatively under divergent selection and might be involved in local adaptation processes in response to the different ecological and environmental gradients experienced by the species.

### 4.1 Population genetic structure

Principal component analyses and Bayesian clustering analyses showed the presence of two well‐defined genetic groups corresponding to Iberian and Canarian populations. The large geographical distance between these two regions (1,700 km), their separation by oceanic water barriers to dispersal, and genetic drift in the small and highly isolated populations from the Canary Islands are probably the main drivers of observed patterns of genetic structure (e.g., Chapuis et al., 2009). According to this last point, Canarian populations presented lower levels of genetic diversity than most Iberian populations (Figure 5a; Table 1) and structure analyses showed that genetic drift after divergence for the cluster corresponding to the Canary Islands was much higher than that estimated for Iberian populations (Pritchard et al., 2000). PCA and hierarchical structure analyses for the two main genetic clusters showed that Canarian populations split into two very well‐defined genetic clusters corresponding to Tenerife and El Hierro Islands, whereas continental populations from Iberian Peninsula presented a much shallower genetic structure and considerable admixture across all analyses. structure analyses for Iberian populations showed the presence of two genetic clusters mostly corresponding to the nonoutbreaking and relatively isolated populations of BERZ and HOYO (Figure 5c). The genetic clusters of these two populations had much higher estimates of genetic drift after divergence than the one representing the rest of Iberian populations, which suggests that their relatively small sizes and/or lower connectivity with other populations are probably the main causes underlying their stronger genetic differentiation. Accordingly, one of these populations (BERZ) had the lowest levels of genetic diversity among all Iberian populations (Table 1). Apart from these two exceptions, we did not find clear differences in spatial patterns of genetic diversity and structure between outbreak and nonoutbreak populations. Levels of genetic differentiation among Iberian populations of *D. maroccanus* (mean *F*_ST_ = 0.067; range = 0.051–0.102) were much lower than those reported in previous SNP‐based studies on nonpest cross‐backed grasshoppers (genus *Dociostaurus*) with narrower distributions such as *D. crassiusculus* (mean *F*_ST_ = 0.129; range = 0.033–0.237; González‐Serna et al., 2018) and *D. hispanicus* (mean *F*_ST_ = 0.189; range = 0.082–0.307; González‐Serna et al., 2019). As expected, levels of genetic differentiation among populations of the Moroccan locust were similar, albeit in some cases sensibly higher, than microsatellite‐based estimates obtained for other locusts and pest grasshoppers sampled at a similar spatial scale (Table A3). However, it should be noted that the different kinds of markers employed (i.e., SNPs versus microsatellites) make difficult the direct comparison of our estimates of genetic differentiation with those obtained in previous studies and, thus, small differences in absolute values must be interpreted with extreme caution (DeFaveri, Viitaniemi, Leder, & Merila, 2013; Ryynanen, Tonteri, Vasemagi, & Primmer, 2007). Overall, our results are in agreement with previous studies on other locust (e.g., Chapuis et al., 2008; Chapuis et al., 2011) and pelagic marine species (e.g., Als et al., 2011; Hoarau, Rijnsdorp, Veer, Stam, & Olsen, 2002) finding very low levels of genetic differentiation and indicate occasional genetic drift and differentiation in nonoutbreaking, solitarious or phase transition populations persisting at low densities in isolated pockets of suitable habitats (Chapuis et al., 2014; Ibrahim et al., 2000).

### 4.2 Past demographic history

Demographic reconstructions for the Moroccan locust using stairway plot revealed the presence of a remarkable genetic bottleneck during the last glacial maximum (~21 ka BP) followed by an abrupt expansion at the onset of the Holocene (Figure 2). Changes in effective population size (*N*_e_) through time did not qualitatively differ among populations, indicating that all of them have experienced parallel demographic histories. Demographic reconstructions for the recent past did not reveal more pronounced expansions in outbreak populations or population bottlenecks in solitarious or phase transition populations. It is also noteworthy that Canarian and Iberian population presented similar demographic profiles, with two main differences: Canarian populations consistently sustained lower effective population sizes over time and experienced less marked demographic declines during the last glacial period than Iberian populations. These results are congruent with the comparatively lower levels of genetic diversity of Canarian populations (Table 1) and compatible with the less severe impact of Pleistocene climatic oscillations at lower latitudes (Fernández‐Palacios et al., 2016; Hewitt, 1999; Snyder, 2016). The inferred demographic trends are also in agreement with fossil records of egg pods of *D. maroccanus* in sediments from the Canary Islands (Meco et al., 2011; Meco, Petit‐Maire, Ballester, Betancort, & Ramos, 2010). Paleontological evidence indicates that the abundance of the species has dramatically oscillated since the end of the Pliocene (3 Ma), with population peaks matching with warm interglacial periods of the Middle and Late Pleistocene (Meco et al., 2010, 2011; Snyder, 2016). The overall good correspondence between peaks of population size and warm periods inferred from both fossil records (Meco et al., 2010, 2011) and genomic data (present study) can be explained by the thermophilic nature of the species and the fact that its development and distribution are limited by low ambient temperatures (Aragón et al., 2013; Aragón & Lobo, 2012; Arias‐Giralda et al., 1997; Benlloch, 1947; Quesada‐Moraga & Santiago‐Álvarez, 2000).

### 4.3 Putative loci under selection and potential for local adaptation

Genome scans based on *F*_ST_ outlier tests revealed that a small portion of the sampled genome (between 0.2% and 1.0%, depending on the dataset and method) is putatively under selection, whereas environmental association analyses (EAA) identified a much larger proportion of SNPs (12%–15%) with allele frequencies correlated with one or more environmental gradients. Beyond these differences in numbers, the specific loci identified by the two methods showed little overlap (Figure 3), which is in agreement with the results obtained by previous RADseq‐based studies (e.g., Dudaniec et al., 2018; Guo et al., 2016). Such differences have been interpreted to be a consequence of the contrasting sensitivities of each approach to deal with the effects of genetic drift and structure and by the better performance of EAA methods to detect weak or polygenic signatures of selection (De Mita et al., 2013; Frichot & François, 2015; de Villemereuil, Frichot, Bazin, François, & Gaggiotti, 2014). A BLAST search in an attempt to identify candidate genes associated with SNPs identified as outliers by the three methods yielded no significant alignment with available sequences at NCBI database (see also Jeffery et al., 2018). This might be explained by the very scarce genomic resources available for grasshoppers, with only one draft genome sequenced so far for a species (*Locusta migratoria*) (Wang et al., 2014) belonging to a different subfamily than the Moroccan locust (Cigliano et al., 2019). Also, the putative signals of selection in certain loci could be a consequence of genetic hitchhiking resulted from linkage disequilibrium between the identified outlier SNPs and nearby nonsequenced genes actually subjected to diversifying selection (Smith & Haigh, 1974). This is particularly relevant considering the extraordinarily large size charactering the genome of grasshoppers, which might have resulted in we have only sampled a relatively small representation of it (Camacho et al., 2015; Wang et al., 2014).

### 4.4 Conclusions and prospects

Overall, our study shows for the first time that populations of the economically important Moroccan locust present a shallow genetic differentiation and little differences in past demography, putative signatures of selection, and contemporary levels of genetic diversity and structure between outbreak and nonoutbreak populations. Outbreaks of Moroccan locust are usually linked to considerable cattle densities and overgrazing (Latchininsky, 1998; Louveaux et al., 1996), and it has been suggested that their frequency might increase in the future favored by global warming (Aragón et al., 2013). Although genetically nondifferentiated populations have often been interpreted as a single panmictic unit (e.g., Hoarau et al., 2002; Schrey, Schrey, Heist, & Reeve, 2008), the homogenizing effects of gene flow do not necessarily indicate demographic dependence or synchrony among populations (Chapuis et al., 2011) and evidence of selective processes inferred in this study suggests that some populations might experience idiosyncratic evolutionary dynamics (e.g., Guo et al., 2016; Pujolar et al., 2014). Future longitudinal and functional genomic studies could help to identify the proximate factors and specific genes underlying the observed putative signatures of selection (e.g., Bakkali & Martín‐Blázquez, 2018; Wang et al., 2014) and determine whether these are temporally stable or if, on the contrary, they are ephemeral and restricted to one or a few generations due to the homogenizing effects of gene flow (Babin et al., 2017; Laporte et al., 2016; Pujolar et al., 2014). The low levels of genetic differentiation among Iberian populations of the Moroccan locust revealed by our analyses indicate that even the application of thousands of SNP markers will not be able to identify the source of nascent outbreak populations at regional scales (see also Chapuis et al., 2009). However, these markers would be useful to determine the origin of eventual waves of long‐distance migrants from remote populations with genotypic differences. The high connectivity among populations of the Moroccan locust within the Iberian Peninsula also points out that the demographic dynamics of the species largely exceed regional and national boundaries. For this reason, and in order to avoid the implementation of local control actions with limited success and high environmental and economic costs, the development of future monitoring and sustainable management programs will require the coordination across the different administration levels and government authorities involved. Overall, the results of this study provide baseline information about some unknown aspects of the biology of the Moroccan locust with implications to develop integrated management practices aimed at reducing the negative impacts of this species that is expected to experience in the near future range expansions and increased outbreak intensity in response to ongoing climate warming and land use alterations (Abrol, 2014; Aragón et al., 2013; Benfekih, Chara, & Doumandji‐Mitche, 2002; Lankau et al., 2011).

## CONFLICT OF INTEREST

The authors declare no conflict of interest.

## AUTHORS' CONTRIBUTIONS

MGS, PJC, and JO conceived the study and collected the samples. MGS and JO designed the study and analyses. MGS performed the laboratory work and analyzed the data guided by JO. MGS and JO led manuscript preparation with input from PJC.

## Supporting information

Supplementary Material

## Data Availability

SNP datasets, input files for structure, PCA, construct, and stairway plot analyses are available for download from the Dryad Digital Repository (https://doi.org/10.5061/dryad.hx3ffbgb2).
