# Supplementary material for: Insights into the neutral and adaptive processes shaping the spatial distribution of genomic variation in the economically important Moroccan locust (Dociostaurus maroccanus)
Source: Ecol Evol. 2020 Mar 31;10(9):3991–4008. doi: 10.1002/ece3.6165 (PMC7244894; doi:10.1002/ece3.6165)
Supplement: Supplementary file 1 — Supplementary Material [file ECE3-10-3991-s001.docx]

**APPENDIX 1**

**CONTENTS**

1. Contributions (factor loadings) of each environmental variable to the different principal components (Table A1)

2. Pairwise *F*_ST_ values (Table A2)

3. Genetic differentiation among populations for different locust and pest grasshopper species (Table A3).

4. Number of reads per individual before and after different quality filtering steps by Stacks (Figure A1)

5. Results of LFMM analyses for three environmental principal components (Figure A2)

6. Results of Bayesian clustering analyses in Structure (Figure A3)

7. Bar plots of Bayesian clustering analyses in Structure using prior population information (Figure A4)

8. Results of spatial clustering analyses in Construct (Figure A5)

9. Bar plots of spatial clustering analyses in Construct (Figure A6)

10. Inferred demographic profiles for populations of Moroccan locust using Stairway plot (Figure A7)

**Table A1** Contributions (factor loadings) of each environmental variable to the different principal components (PC1, PC2 and PC3). Values > 0.8 and < -0.8 are highlighted in grey.

| Environmental variables | PC1 | PC2 | PC3 |
| --- | --- | --- | --- |
| Annual Mean Temperature | 0.855 | 0.392 | -0.289 |
| Mean Diurnal Range (Mean of monthly (max temp - min temp)) | -0.478 | 0.837 | -0.135 |
| Isothermality (BIO2/BIO7) (* 100) | 0.085 | -0.874 | -0.262 |
| Temperature Seasonality (standard deviation *100) | -0.438 | 0.896 | -0.050 |
| Max Temperature of Warmest Month | 0.167 | 0.975 | -0.116 |
| Min Temperature of Coldest Month | 0.940 | -0.299 | -0.074 |
| Temperature Annual Range (BIO5-BIO6) | -0.420 | 0.899 | -0.050 |
| Mean Temperature of Wettest Quarter | 0.198 | -0.110 | -0.835 |
| Mean Temperature of Driest Quarter | 0.404 | 0.875 | -0.242 |
| Mean Temperature of Warmest Quarter | 0.458 | 0.848 | -0.240 |
| Mean Temperature of Coldest Quarter | 0.950 | -0.187 | -0.195 |
| Annual Precipitation | -0.298 | 0.222 | 0.902 |
| Precipitation of Wettest Month | 0.262 | -0.423 | 0.793 |
| Precipitation of Driest Month | -0.955 | -0.094 | -0.121 |
| Precipitation Seasonality (Coefficient of Variation) | 0.812 | -0.450 | 0.341 |
| Precipitation of Wettest Quarter | 0.264 | -0.386 | 0.867 |
| Precipitation of Driest Quarter | -0.965 | 0.063 | -0.174 |
| Precipitation of Warmest Quarter | -0.947 | -0.061 | -0.202 |
| Precipitation of Coldest Quarter | 0.380 | -0.133 | 0.910 |

**Table A2** Pairwise *F*_ST_ values. Upper diagonal shows *F*_ST_ values for all populations and lower diagonal present *F*_ST_ values for analyses restricted to populations from the Iberian Peninsula.

|  | TRAB | SAND | SALA | ALHA | CACE | BERZ | CAST | HOYO | TIRE | CORN | ALCU | BELA | BONI | FELI | SANT | ESPA | JUMI | CARA | ALPU | TENE | HIER |
| --- | --- | --- | --- | --- | --- | --- | --- | --- | --- | --- | --- | --- | --- | --- | --- | --- | --- | --- | --- | --- | --- |
| TRAB |  | 0.052 | 0.061 | 0.053 | 0.053 | 0.078 | 0.052 | 0.058 | 0.054 | 0.060 | 0.054 | 0.062 | 0.061 | 0.063 | 0.062 | 0.064 | 0.061 | 0.051 | 0.058 | 0.084 | 0.078 |
| SAND | 0.052 |  | 0.060 | 0.054 | 0.051 | 0.079 | 0.051 | 0.058 | 0.054 | 0.061 | 0.054 | 0.061 | 0.061 | 0.062 | 0.061 | 0.063 | 0.061 | 0.051 | 0.056 | 0.083 | 0.077 |
| SALA | 0.061 | 0.061 |  | 0.063 | 0.062 | 0.096 | 0.060 | 0.069 | 0.066 | 0.077 | 0.065 | 0.076 | 0.073 | 0.078 | 0.077 | 0.079 | 0.077 | 0.061 | 0.065 | 0.101 | 0.095 |
| ALHA | 0.054 | 0.055 | 0.064 |  | 0.053 | 0.078 | 0.052 | 0.061 | 0.057 | 0.063 | 0.054 | 0.063 | 0.064 | 0.067 | 0.064 | 0.065 | 0.065 | 0.054 | 0.057 | 0.085 | 0.081 |
| CACE | 0.053 | 0.052 | 0.063 | 0.053 |  | 0.079 | 0.054 | 0.057 | 0.056 | 0.063 | 0.056 | 0.064 | 0.063 | 0.064 | 0.064 | 0.064 | 0.062 | 0.051 | 0.055 | 0.084 | 0.078 |
| BERZ | 0.079 | 0.079 | 0.097 | 0.079 | 0.079 |  | 0.078 | 0.086 | 0.082 | 0.101 | 0.085 | 0.099 | 0.092 | 0.097 | 0.101 | 0.102 | 0.096 | 0.077 | 0.084 | 0.123 | 0.116 |
| CAST | 0.052 | 0.051 | 0.060 | 0.053 | 0.054 | 0.079 |  | 0.056 | 0.056 | 0.062 | 0.056 | 0.064 | 0.062 | 0.063 | 0.063 | 0.062 | 0.062 | 0.052 | 0.056 | 0.083 | 0.078 |
| HOYO | 0.059 | 0.059 | 0.070 | 0.062 | 0.057 | 0.087 | 0.057 |  | 0.061 | 0.068 | 0.060 | 0.068 | 0.069 | 0.071 | 0.069 | 0.072 | 0.069 | 0.057 | 0.063 | 0.090 | 0.084 |
| TIRE | 0.055 | 0.055 | 0.066 | 0.058 | 0.056 | 0.083 | 0.056 | 0.061 |  | 0.067 | 0.059 | 0.068 | 0.067 | 0.070 | 0.067 | 0.068 | 0.068 | 0.055 | 0.058 | 0.088 | 0.082 |
| CORN | 0.060 | 0.061 | 0.078 | 0.063 | 0.063 | 0.101 | 0.062 | 0.068 | 0.067 |  | 0.067 | 0.079 | 0.074 | 0.080 | 0.080 | 0.081 | 0.078 | 0.061 | 0.064 | 0.102 | 0.096 |
| ALCU | 0.055 | 0.054 | 0.066 | 0.055 | 0.056 | 0.085 | 0.056 | 0.060 | 0.060 | 0.067 |  | 0.068 | 0.066 | 0.068 | 0.069 | 0.068 | 0.066 | 0.054 | 0.059 | 0.088 | 0.082 |
| BELA | 0.063 | 0.061 | 0.077 | 0.064 | 0.064 | 0.099 | 0.065 | 0.068 | 0.068 | 0.080 | 0.069 |  | 0.077 | 0.081 | 0.081 | 0.080 | 0.079 | 0.060 | 0.067 | 0.102 | 0.095 |
| BONI | 0.062 | 0.062 | 0.074 | 0.065 | 0.064 | 0.093 | 0.062 | 0.070 | 0.068 | 0.075 | 0.066 | 0.077 |  | 0.080 | 0.077 | 0.076 | 0.077 | 0.061 | 0.067 | 0.099 | 0.093 |
| FELI | 0.063 | 0.063 | 0.078 | 0.068 | 0.064 | 0.098 | 0.064 | 0.072 | 0.070 | 0.080 | 0.068 | 0.082 | 0.080 |  | 0.082 | 0.081 | 0.083 | 0.063 | 0.068 | 0.104 | 0.098 |
| SANT | 0.063 | 0.061 | 0.077 | 0.065 | 0.064 | 0.101 | 0.064 | 0.069 | 0.068 | 0.080 | 0.069 | 0.082 | 0.078 | 0.083 |  | 0.079 | 0.080 | 0.061 | 0.067 | 0.103 | 0.096 |
| ESPA | 0.064 | 0.063 | 0.080 | 0.066 | 0.064 | 0.102 | 0.063 | 0.073 | 0.068 | 0.081 | 0.068 | 0.080 | 0.077 | 0.081 | 0.080 |  | 0.079 | 0.062 | 0.070 | 0.105 | 0.097 |
| JUMI | 0.062 | 0.061 | 0.078 | 0.066 | 0.063 | 0.097 | 0.062 | 0.069 | 0.069 | 0.079 | 0.067 | 0.080 | 0.078 | 0.083 | 0.081 | 0.079 |  | 0.062 | 0.065 | 0.102 | 0.096 |
| CARA | 0.051 | 0.051 | 0.061 | 0.055 | 0.052 | 0.077 | 0.052 | 0.058 | 0.055 | 0.062 | 0.054 | 0.061 | 0.062 | 0.064 | 0.062 | 0.063 | 0.062 |  | 0.054 | 0.082 | 0.078 |
| ALPU | 0.059 | 0.057 | 0.065 | 0.058 | 0.056 | 0.085 | 0.056 | 0.064 | 0.059 | 0.065 | 0.060 | 0.068 | 0.068 | 0.068 | 0.068 | 0.070 | 0.065 | 0.055 |  | 0.089 | 0.082 |
| TENE |  |  |  |  |  |  |  |  |  |  |  |  |  |  |  |  |  |  |  |  | 0.060 |
| HIER |  |  |  |  |  |  |  |  |  |  |  |  |  |  |  |  |  |  |  |  |  |

**Table A3** Genetic differentiation among populations for different locust and pest grasshopper species. Table shows the area of study, the number of sampled populations (*n*), mean and range of pairwise *F*_ST_ values between populations, type and number of markers employed, and reference. Asterisks mark geographic areas comparable in size to our sampling area within the Iberian Peninsula.

| Species | Geographic area | *n* | *F*_ST_ (mean) | *F*_ST_ (range) | Marker (number) | Reference |
| --- | --- | --- | --- | --- | --- | --- |
| *Locusta migratoria* | Europe, Africa, Asia, Australia | 25 | 0.0740 | -0.002/0.2650 | Microsatellites (14) | Chapuis et al., 2008^a^ |
| *Locusta migratoria* | Europe* | 4 | 0.0450 | 0.0330/0.0660 | Microsatellites (14) | Chapuis et al., 2008^a^ |
| *Locusta migratoria* | Africa (excluding Madagascar) | 5 | 0.0060 | 0.0010/0.0150 | Microsatellites (14) | Chapuis et al., 2008^a^ |
| *Chortoicetes terminifera* | Australia | 12 | -0.0002 | -0.0071/0.0057 | Microsatellites (7) | Chapuis et al., 2011^b^ |
| *Chortoicetes terminifera* | Eastern Australia | 6 | -0.0003 | -0.0041/0.0052 | Microsatellites (7) | Chapuis et al., 2011^b^ |
| *Chortoicetes terminifera* | South-Western Australia* | 5 | -0.0018 | -0.0045/0.0032 | Microsatellites (7) | Chapuis et al., 2011^b^ |
| *Schistocerca gregaria* | North Africa, Pakistan | 23 | 0.0196 | -0.0050/0.2340 | Microsatellites (23) | Chapuis et al., 2014^c^ |
| *Schistocerca gregaria* | Mauritania* | 9 | 0.0049 | -0.0030/0.0270 | Microsatellites (23) | Chapuis et al., 2014^c^ |
| *Schistocerca gregaria* | Chad* | 7 | 0.0027 | -0.0050/0.0140 | Microsatellites (23) | Chapuis et al., 2014^c^ |
| *Calliptamus barbarus* | South-Western Europe | 13 | 0.0349 | 0.0010/0.0820 | Microsatellites (7) | Blanchet et al., 2012^d^ |
| *Calliptamus barbarus* | South-Eastern France, North-Eastern Spain* | 11 | 0.0247 | 0.0010/0.0620 | Microsatellites (7) | Blanchet et al., 2012^d^ |
| *Calliptamus italicus* | South-Eastern France | 5 | 0.0193 | 0.0020/0.0460 | Microsatellites (6) | Blanchet et al., 2012^d^ |
| *Calliptamus wattenwylianus* | France, Spain, Morocco, Algeria | 10 | 0.0088 | -0.0100/0.0370 | Microsatellites (3) | Blanchet et al., 2012^d^ |
| *Calliptamus wattenwylianus* | South-Eastern France, North-Eastern Spain* | 8 | 0.0114 | -0.0020/0.0370 | Microsatellites (3) | Blanchet et al., 2012^d^ |
| *Dociostaurus maroccanus* | Iberian Peninsula, Canary Islands | 21 | 0.0713 | 0.0510/0.1230 | SNPs (40,179) | This study |
| *Dociostaurus maroccanus* | Iberian Peninsula* | 19 | 0.0669 | 0.0510/0.1020 | SNPs (42,114) | This study |

^a^ Chapuis et al., 2008, *Molecular Ecology*, 17(16), 3640-3653.

^b^ Chapuis et al., 2011, *Proceedings of the Royal Society of London. Series B: Biological Sciences*, 278(1721), 3152-3160.

^c^ Chapuis et al., 2014, *Molecular Ecology*, 23(7), 1749-1763.

^d^ Blanchet et al., 2012, *European Journal of Entomology*, 109(3), 445-455.

**
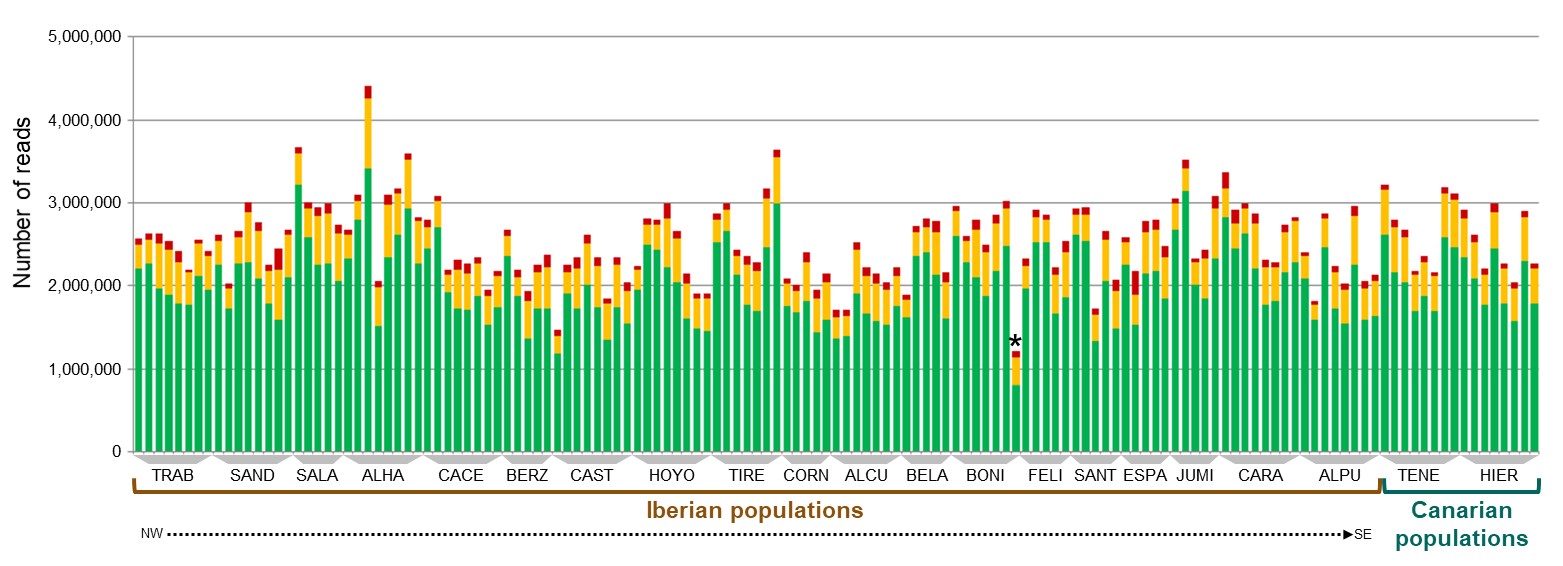
Figure A1** Number of reads per individual before and after different quality filtering steps by Stacks. The total height of the bars represents the total number of raw reads obtained for each individual. Within each bar, the dark red color represents the reads that were discarded by *process_radtags* due to low quality, adapter contamination or ambiguous barcode and orange color represents the reads that were discarded by *ustacks* after filtering out repetitive elements and reads that did not comply the different criteria required to create a “stack”. Green color represents the number of retained reads used to identify homologous loci. The individual with an asterisk was removed for subsequent analyses (< 1,000,000 retained reads). Populations are labelled using the same codes presented in Table S1.

**Figure A2** Results of LFMM analyses for three environmental principal components (PC1, PC2 and PC3). Histograms show the frequency of loci with different *p*-values for each environmental principal component tested (PC1, PC2 and PC3). Analyses were performed for (A, B, C) all populations and (D, E, F) only considering populations from the Iberian Peninsula. Grey bars indicate unadjusted *p*-values and green bars indicate *p*-values adjusted with the genomic inflation factor (λ) that is indicated in each panel.


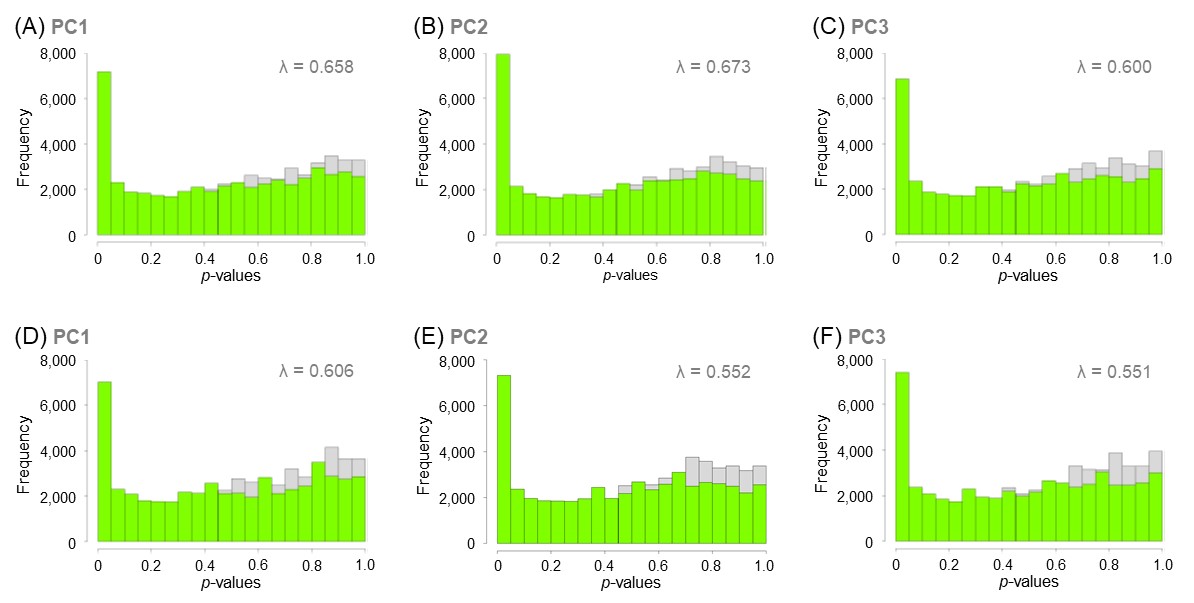


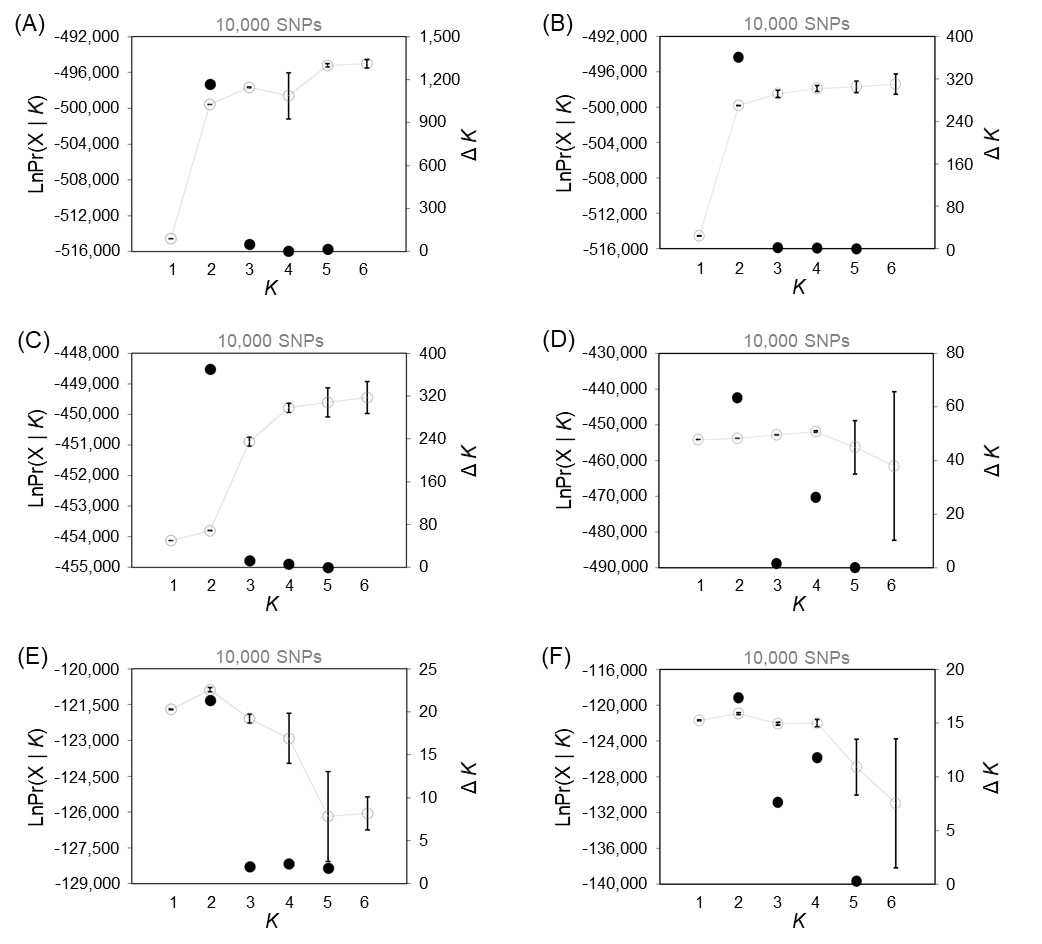
**Figure A3** Results of Bayesian clustering analyses in Structure (A, C, E) not considering and (B, D, F) considering prior population information. Analyses were performed for (A, B) all populations and only considering populations from (C, D) the Iberian Peninsula or (E, F) the Canary Islands. Panels show the mean (± SD) log probability of the data (LnPr (X|*K*) over 10 best runs (left *y*-axes, open dots, and error bars) for each value of *K* and the magnitude of Δ*K* (right *y*-axes, black dots). The number of loci used in the different analyses is indicated in each panel.

**Figure A4** (A-B) Genetic diversity and (C) results of Structure analyses considering prior population information for the studied populations of Moroccan locust. Panel (A) shows nucleotide diversity (π) for each population calculated in stacks for all positions (polymorphic and non-polymorphic). Panel (B) shows standardized multilocus heterozygosity (sMLH) of each individual, with values below the lowest 10^th^ percentile shown in red. (C) Bar plots of Structure analyses show the individual’s probabilities of membership to each inferred genetic cluster for different *K* values. Each individual is represented by a vertical bar, which is partitioned into *k* coloured segments showing the individual’s probability of belonging to the cluster with that colour. Thin vertical black lines separate individuals from different populations. Structure analyses were performed at different hierarchical levels, analysing all populations together and Iberian (brownish) and Canarian (greenish) populations separately. Populations with an asterisk indicate pest outbreaks during the sampling year. Population codes are described in Table 1.

**Figure A5** (A) Cross-validation results for data simulated under *K* = 1-6 and the spatial Construct model for Iberian populations of Moroccan locust. Plot shows mean predictive accuracy and 95% confidence intervals for each value of *K*. (B) Layer/cluster contributions (i.e., how much each layer/cluster contributes to total covariance) for spatial models run from *K* = 1 to *K* = 6.

**
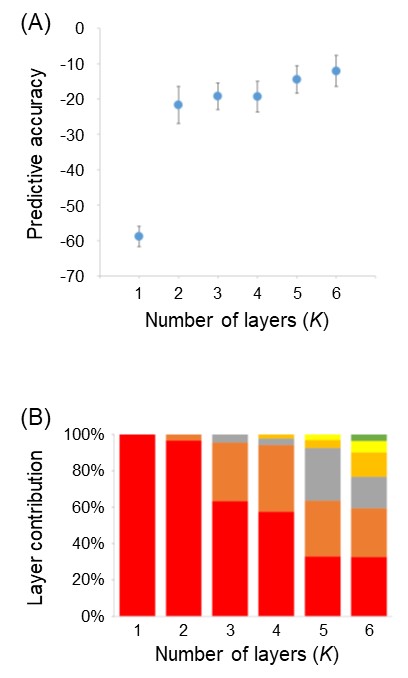
**

**Figure A6** Results of spatial analyses in Construct for Iberian populations of Moroccan locust. Bar plots show the individual’s probabilities of membership to each inferred genetic cluster for different *K* values. Each individual is represented by a vertical bar, which is partitioned into *k* coloured segments showing the individual’s probability of belonging to the cluster with that colour. Thin vertical black lines separate individuals from different populations. Populations with an asterisk indicate pest outbreaks during the sampling year. Population codes are described in Table 1.

**Figure A7** Inferred demographic profiles for populations of Moroccan locust using Stairway plot**.** Within in each panel, the red line shows the median estimate of effective population size *(N*_e_) over time, assuming a mutation rate of 2.8 × 10^-9^ and 1-year generation time. Grey lines represent confidence intervals (CI) obtained in Stairway plot: thick grey lines are 97.5% and 2.5% percentiles, and thin grey lines are 87.5% and 12.5% percentiles, respectively. Populations with an asterisk indicate pest outbreaks during the sampling year. Population codes are described in Table S1 and name colours are the same as in Figure 1.
